# FBS-based cryoprotective compositions for effective cryopreservation of gut microbiota and key intestinal microorganisms

**DOI:** 10.1186/s13104-024-06836-2

**Published:** 2024-06-19

**Authors:** Lyubov V. Zalomova, Eugeny E. Fesenko

**Affiliations:** Institute of Cell Biophysics PSCBR RAS, Moscow Region, Institutskaya Str., 3, Pushchino, 142290 Russia

**Keywords:** Cryopreservation, Gut microbiota, Bacterial cultures, Fetal bovine serum

## Abstract

**Objective:**

The need for innovative techniques to preserve microbiota for extended periods, while maintaining the species composition and quantitative balance of the bacterial community, is becoming increasingly important. To address this need, we propose an efficient approach to cryopreserve human gut microbiota using a two-component cryoprotective composition comprising fetal bovine serum (FBS) and 5% dimethyl sulfoxide (DMSO). Fetal serum is a commonly utilized component in the freezing media for eukaryotic cells, however, its effects on prokaryotic cells have not been extensively researched.

**Results:**

In our study, we demonstrated the high efficiency of using a two-component cryoprotective medium, FBS + 5% DMSO, for cryopreservation of human gut microbiota using three different methods. According to the obtained results, the intact donor microbiota was preserved at a level of 85 ± 4% of the initial composition based on fluorescent analysis using the LIVE/DEAD test. No differences in survival were observed when comparing with pure DMSO and FBS media. The photometric measurement method for growth of aerobic bacteria *(A. johnsoni),* facultative anaerobes *(E. coli, E. faecalis),* microaerophilic *(L. plantarum),* and obligate anaerobic bacterial cultures *(E. barkeri, B. breve)* also demonstrated high viability rates of 94–98% in the two-component protective medium, reaching intact control levels. However, for anaerobic microflora representatives, serum proved to be a more suitable cryoprotectant. Also, we demonstrated that using cryoprotective media with 50–75% FBS content is enough to preserve a significant level of bacterial cell viability, from an economic standpoint.

## Introduction

Maintaining a balanced and diverse microbiota in the human gut is crucial for preventing various diseases such as diabetes, obesity, and autoimmune disorders [1–3]. Cryopreservation of human gut microbiota is essential for preserving symbiotic associations of the intestine for a long time, which can be used as a source of personalized probiotics (autoprobiotics) and for safe and efficient transplantation to restore the microbiota balance [4].

However, cryopreservation is challenging due to the diversity of properties of symbiotic organisms and their different cryotolerance [5]. In our 2019 review [6], we synthesized the latest research on cryopreservation methods for gut microbiota. Most studies attempted to optimize protocols for specific bacterial cultures, with around 70% describing the use of 10–20% glycerol or DMSO and the remaining focusing on non-penetrating cryoprotectors. The most common cryopreservation regimens involved rapid freezing in liquid nitrogen (− 196 °C) which can be followed by transfer to an ultralow temperature freezer (− 80 °C), or slow freezing by direct transfer to a − 80 °C freezer. A few recent studies also focused on using single main cryoprotectants such as 30% glycerol [7], 10–20% trehalose, betaine, and proline, as well as PBS-based solutions [8], polymers (PEG, PVA) [9]. While these methods have shown some success, we believe that utilizing multicomponent media for freezing is a more promising approach to increase the efficiency of cryopreservation.

In this study, we propose an efficient approach using a two-component cryoprotective composition comprising fetal bovine serum (FBS) and 5% dimethyl sulfoxide (DMSO) for cryopreserving human gut microbiota. We have previously investigated the cryoprotective properties of different compounds (DMSO, glycerol, ethylene glycol, gelatine, polyethylene glycol (PEG), FBS) and found that FBS and 5% DMSO demonstrated the best cryoprotective properties [10, 11]. Fetal serum is a commonly utilized component in the freezing media for eukaryotic cells, however, its effects on prokaryotic cells have not been extensively researched [12–14].

In this expanded survey, we show the efficiency of this two-component composition during cryopreservation of human whole intestinal microbiota and key representatives of intestinal microflora.

## Materials and methods

### Objects

Bacterial strains *Escherichia coli* К-802*, Enterococcus faecalis* B-1578*, Lactobacillus plantarum* B-578*, Bifidobacterium breve* DSM 20213*, Eubacterium barkeri* B-1775*, Acinetobacter johnsoni* B-3187 were purchased from All-Russian Collection of Microorganisms IBPM RAS. The study utilized whole intestinal microbiota that was obtained from a pool of 15 healthy male and female donors between the ages of 18 to 40 years old. Prior to donation, the donors provided informed consent for their microbiota to be used in the study approved by the Commission on Biosafety and Bioethics ICB PSCBR RAS (Approval ID 3/092021 date 08.09.2021).

### Preparation of bacterial suspensions

The bacterial suspensions of monocultures used in this study were prepared by diluting them either in a cryoprotective medium or in 0.9% NaCl to achieve a final concentration of 10^7^ cells/ml for microtiter plate-based cultivation. Fecal samples weighing 0.1 g were diluted in 1 ml of sterile 0.9% NaCl and thoroughly resuspended to form a homogenous suspension. The suspension was centrifuged for 5 min at 1200 rpm (4230 g) on MiniSpin centrifuge (Eppendorf, Germany), and then the supernatant with microbial cells was collected. Then it was centrifuged at 10,000 rpm (12,200 g) for 10 min. and the cell pellet obtained was resuspended in 1 ml of 0.9% NaCl. The concentration of cells in the resulting suspension was counted using a Goryayev chamber, and the bacterial suspension was used at a final concentration of 10^8^ cells/ml. Such a cell concentration was chosen in accordance with the protocol for conducting fluorescent analysis using the LIVE/DEAD test.

### Cryopreservation

5% DMSO (AppliChem, Germany)/0.9% NaCl, 5% DMSO/10–95% FBS (Biosera, France)/0.9% NaCl and 10–100% FBS /0.9% NaCl were used as a cryoprotectors. The solution of 0.9% NaCl was used for cryopreservation of the control samples.

Cryopreservation was carried out by immersing the cryogenic storage vials (1 ml) (Corning, USA) with bacterial suspensions (1 ml) in liquid nitrogen. Pipetting of the cell suspensions into the vials was carried out under nitrogen flow to provide anaerobic conditions. The storage period comprised 4 days. After this period, the samples were thawed in water bath at 37 °C for 5 min. Cryoprotectors were removed by centrifugation and washing.

### Viability tests

#### Vital staining analysis

LIVE/DEAD BacLight Bacterial Viability Kit 7012 (Molecular Probe, USA) was used to evaluate live bacterial cells. The staining was performed according to the manufacturer's instruction. Fluorescence intensity was measured on a plate photometer (FilterMax F5, USA).

#### Microbiological cultivation

*Escherichia coli* cells (10^7^ cells/ml) were cultivated in Petri dishes (Thermo Scientific, USA) on solid selective Endo agar medium (CCM SRCAM), Obolensk, Russia) at constant temperature (37 °C) in a thermostat Heratherm IMC 18 (Thermo Scientific, USA) for 24 h in aerobic conditions. To assess the viability of the bacteria, we performed a colony-forming unit (CFU) count on a range of dilutions. The resulting values were then converted into percentages relative to the initial cell concentration (100%).

#### Optical density registration

Microtiter plate photometer (Biotek Synergy H1, USA) connected to nitrogen-carbon dioxide mixture supply was used in the experiment. After thawing, the suspended bacterial monocultures *Acinetobacter johnsoni, Eubacterium barkeri* and *Enterococcus faecalis* were supplied with 150 μL fresh thioglycolate broth. For the *Bifidobacteria*, special medium was used (MRS-1 medium), for the *Lactobacteria*—MRS-1-medium and for *E. coli*—medium Luria Bertani (LB).

All bacterial suspensions were subsequently transferred into a 96-well microtiter plate in 100 μL aliquots. The following parameters of cultivation were set in Gene 5 3.03: T = 30–37 °C (depending on species/strain), continuous shaking “ON”, 1 h interval between optical density measurements (ƛ = 600 nm), 24 h cell growth time. Cultivation of obligate anaerobes (*B. breve* and *E. barkeri)* and microaerophiles *(L. plantarum*) was conducted under nitrogen-carbon dioxide atmosphere (N_2_—80%, CO_2_—20%).

### Statistical data analysis

Statistical data analysis was performed in Sigma Plot 14.0 (Systat Software, Inc, Canada). The statistical significance of the differences between the groups was assessed according to Mann–Whitney U-test.

## Results

### The viability of *Escherichia* coli after cryopreservation using 5% DMSO, 100% FBS, and a combination of 5% DMSO and 95% FBS: comparative analysis of 3 viability tests

**Fig. 1:**
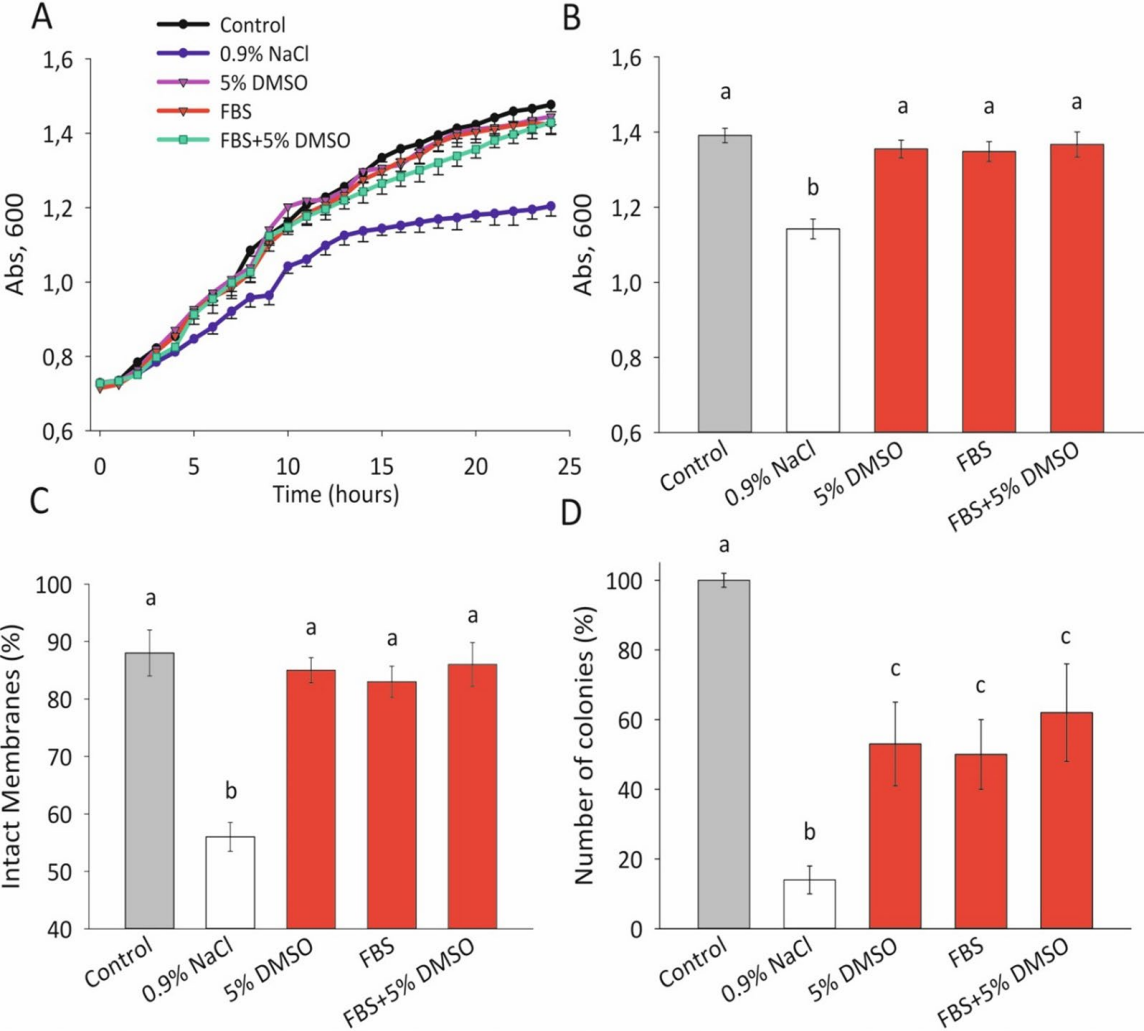
Viability of *Escherichia coli* after cryopreservation (4 days at − 196 °C) in the media 5% DMSO, 100% FBS, and a combination of 5% DMSO and 95%FBS by three different methods: microtiter plate-based cultivation (**A**), (**B**), (n = 3); fluorescent LIVE/DEAD assay (**C**) (n = 10), conventional microbiological cultivation (**D**), (n = 10). Abs—optical density (600 nm). Columns marked with the same letter are not significantly different at p < 0.05, according to the Mann–Whitney nonparametric criterion

Growth curves of *Escherichia coli* frozen in 5% DMSO, 100% FBS, 5% DMSO + 95% FBS did not actually differ from intact control (no freezing) (Fig. 1A. In addition, diagram 1B (Fig. 1B illustrates the viability results of this culture after 24 h of growth in a microtiter plate. It is worth noting that there were no significant differences in the optical density values among the 5% DMSO, 100% FBS and 5% DMSO + 95% FBS groups. The same image was recorded using the LIVE-DEAD fluorescent assay to evaluate viability (Fig. 1C. The conventional microbiological cultivation on solid media (Fig. 1D revealed a conservation degree of approximately 60% for the *Escherichia coli* samples compared to the intact control. No differences were found among cryoprotective composition groups using the microbiological cultivation as well as previous two methods.

### The viability of five bacterial cultures and the whole human gut microbiota after cryopreservation using 5% DMSO, 100% FBS, and a combination of 5% DMSO and 95% FBS

**Fig. 2:**
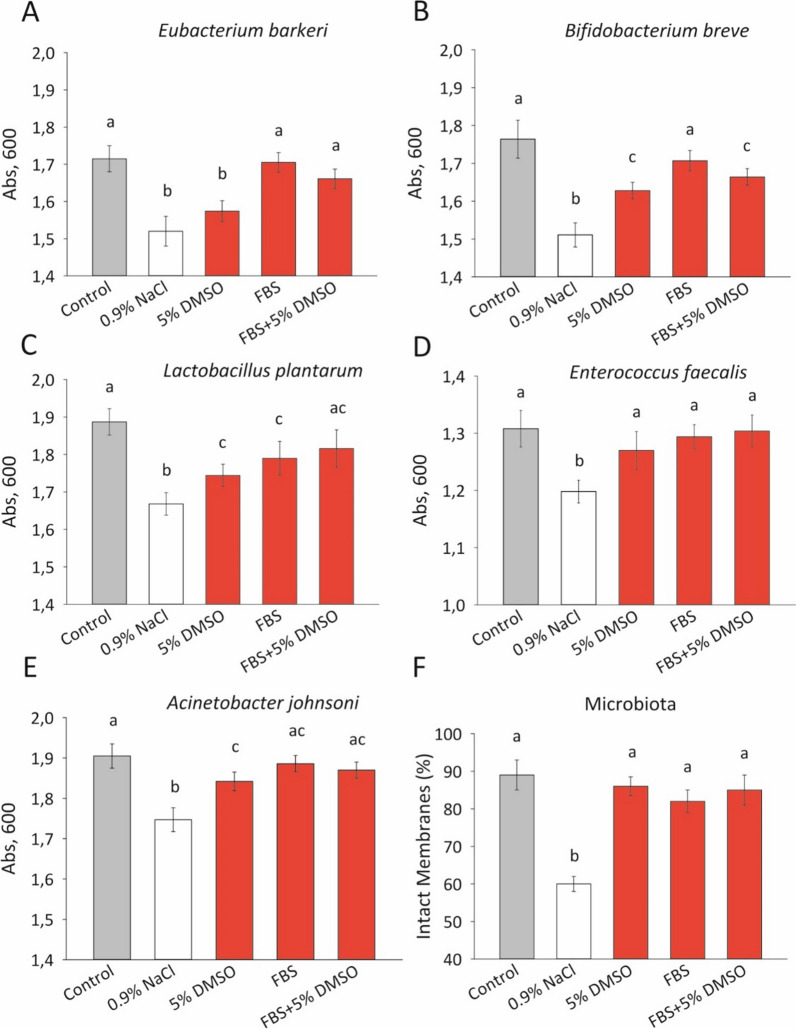
Viability of five bacterial cultures (microtiter plate-based cultivation, n = 3) and whole human gut microbiota (LIVE/DEAD assay, n = 15) after cryopreservation (4 days at − 196 °C, 24 h of growth) in the media 5% DMSO, 100% FBS, and a combination of 5% DMSO and 95% FBS. Abs – optical density (600 nm). **A** *Eubacterium barkeri,* **B** *Bifidobacterium breve,* **C** *Lactobacillus plantarum,* **D** *Enterococcus faecalis,* **E** *Acinetobacter johnsoni,* **F** whole microbiota. Columns marked with the same letter are not significantly different at p < 0.05, according to the Mann–Whitney nonparametric criterion

The microtiter plate-based cultivation study found that the viability of bacterial cells after cryopreservation was maintained at an intact control level only for *E. faecalis* and the entire microbiota (LIVE-DEAD analysis) when 5% DMSO was used (Fig. 2) However, the viability of bacterial cells was comparable to the control in all experiments, except for microaerophilic *L. plantarum*, when 100% FBS was used. Additionally, a combination of 5% DMSO and 95% FBS also maintained the viability of bacterial cells at the control level in all experiments, except for the obligate anaerobe *B. breve.*

### The viability of six bacterial cultures after cryopreservation in media with different

#### FBS concentration

**Fig. 3:**
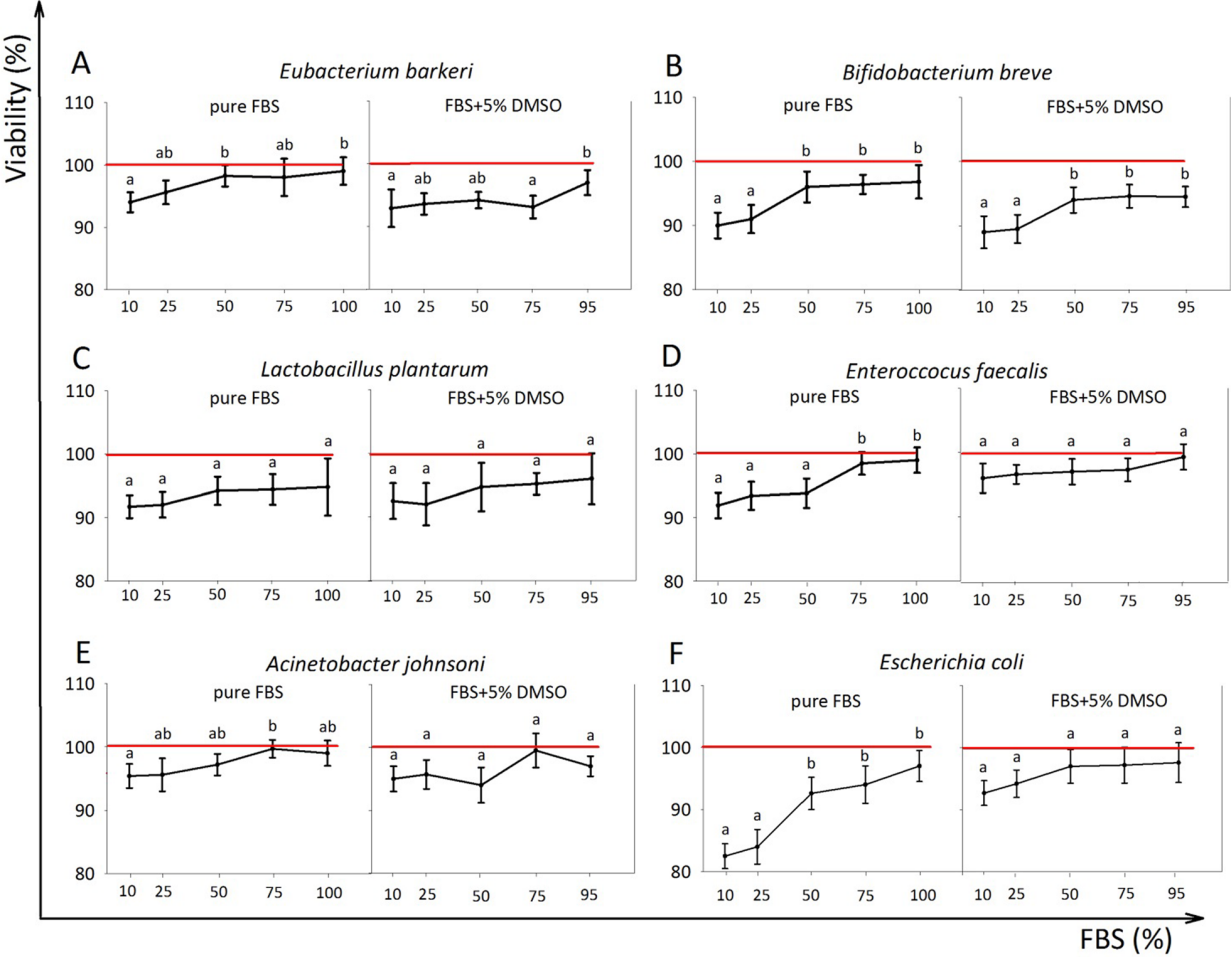
The impact of FBS concentrations on the survival of six bacterial cultures after cryopreservation (4 days at − 196 °C) (n = 3) in pure FBS and FBS + 5% DMSO mixtures. Values are normalized to intact control level (100%). **A** *Eubacterium barkeri* **B***Bifidobacterium breve,*** C** Lactobacillus* plantarum,* **D** *Enterococcus faecalis,* **E** *Acinetobacter johnsoni,* **F** *Escherichia coli.* Points marked with the same letter are not significantly different at p < 0.05, according to the Mann–Whitney nonparametric criterion

The microtiter plate-based cultivation results showed that there was no significant difference in bacterial viability after cryopreservation between the groups with 75 and 100% FBS for all six cultures, and between 50, 75, and 100% FBS for five cultures, as well as between 10 and 100% of FBS for L. plantarum (Fig. 3). For four out of six cultures, any percentage of FBS was suitable when combined with 5% DMSO, with no significant differences in bacterial viability after cryopreservation in LN. For the obligate anaerobe *Bifidobacterium breve*, there was no significant difference in viability between the 50, 75, and 95% FBS groups, while for the also anaerobic *Eubacterium barkeri*, the 95% FBS group showed the best results.

## Discussion

The comparative analysis of 3 viability tests demonstrated a strong correlation between each method of estimating bacterial culture viability, indicating that all three methods can be utilized independently to accurately assess the state of the culture. The viability levels obtained by conventional cultivation were observed to be approximately 60% compared to the intact control, which may be attributed to the limitations of traditional method.

The obtained plate-based cultivation diagrams indicate that *Enterococcus faecalis*, a facultative aerobe, and *Lactobacillus plantarum*, a microaerophile, exhibited an increasing trend in viability levels with 5% DMSO added to FBS compared to pure serum. This allowed the *L. plantarum* to attain intact control levels, confirming the efficacy of using a two-component mixture to enhance cryopreservation reliability. Conversely, the viability of obligate anaerobes *Eubacterium barkeri* and *Bifidobacterium breve* tended to decrease in 5% DMSO/FBS media. Notably, the preservation level in pure serum was significantly higher for *B. breve* than in 5% DMSO or 5% DMSO/95% FBS. For the investigated anaerobes, serum proved to be a more suitable cryoprotective agent while its presence in the two-component mixture mitigated the negative (toxic) effects of DMSO, noted by some researchers [7]. There was no correlation found between viability and cell wall composition (Gram-positive/Gram-negative). The preservation degree of whole microbiota assessed by fluorescent LIVE/DEAD analysis was around 81–89% with no differences between three media due to the averaging effect in the presence of many bacterial species.

Our analysis showed that both variants—pure FBS and 5% DMSO/95% FBS—demonstrated high bacterial survival rates. However, reducing the serum concentration had a negative impact on bacterial cell survival. We found that in order to maintain a high level of bacterial cell survival, it is not advisable to reduce the serum concentration to less than 75% for pure FBS, and less than 50% for the combination of 5% DMSO and 95% FBS. The limited studies that have utilized serum for bacterial cell preservation did not explore the concentration range suggested by our analysis. For instance, Oskouei et al. employed a 10% serum component in various three-component media for preserving *H. pylori* for one month at − 80 °C [13]. Kirkham et al. used a 20% serum concentration for preserving pathogenic *H. influenzae* for up to eight weeks at − 80 °C [14]. More recently, Farha et al. utilized a combination of 40% FBS, 10% DMSO, and bovine milk for preserving *M. bovis* for up to 16 weeks at − 80 °C [12]. It's worth noting that FBS is not toxic to cells and contains growth factors and biologically active compounds that aid in bacterial recovery after thawing. Due to its relatively high cost, it may be reasonable to consider cryoprotective media with 50%-75% FBS content from an economic standpoint.

An extensive review of publications on cryopreservation of microorganisms revealed that DMSO and glycerol solutions are commonly utilized at concentrations ranging from 10 to 20% [6]. A notable drawback of using cryoprotectants at these concentrations is their toxicity to cells. This toxicity was substantiated by our experimental findings from fluorescence testing and cytotoxicity studies [10], which demonstrated that bacterial cells were better preserved when protected with 5% DMSO or 5% glycerol. According to the recent results obtained by bulk 16S rRNA sequencing [15], 5% DMSO in combination with Cary Blair medium at − 80 °C provided the best survival of various genera of microorganisms, as well as their taxonomic diversity. Interestingly, the authors of [15] highlight that a sample selection strategy based on physicochemical gradients could be a significant factor in maximizing the recovery of a diverse range of bacterial taxa from human stool samples.

The findings conducted by Li et al*.* revealed that utilizing a combination of − 80 °C/liquid nitrogen deep cryopreservation and 10% glycerol proved to be the most efficient method for preserving stool samples, ensuring their viability for extended periods of at least 12 months [16]. This preservation approach rendered the microbiota of the samples suitable for a diverse array of subsequent experimental studies and analyses, including 16S rRNA sequencing. The authors exclusively employed glycerol concentrations of 10% in their research; however, we believe that utilizing a 5% glycerol solution may enhance the survival of various bacterial taxa even better.

## Conclusion

Thus we strongly believe that the proposed cryoprotective compositions offer superior cryoprotective potential compared to other variants of cryoprotective media, especially those based on high concentrations of glycerol or DMSO. The use of two equally effective cryoprotective agents in combination significantly increases the likelihood of successful cryopreservation of the microbial community as a whole. To further enhance the cryoprotective potential of the studied composition, non-penetrating cryoprotectors such as high molecular weight compounds (PEG, PVA) [9] or trehalose could be considered [8]. Since our work has limitations related to the use of pure cultures, we plan to create artificial mixes of different bacterial species in future experiments that will better reflect the application of cryoprotective agents to them.

## Data Availability

The datasets used and/or analysed during the current study are available from the corresponding author on reasonable request.

## Abbreviations

CFU: Colony forming unit
DMSO: Dimethyl sulfoxide
FBS: Fetal bovine serum
PBS: Phosphate buffered saline
PEG: Polyethylene glycol
PVA: Polyvinyl alcohol
